# Evaluating Problematic Smartphone Use Among Chinese Primary School Students Using SABAS: An IRT and Network Analysis

**DOI:** 10.1002/mpr.70016

**Published:** 2025-04-01

**Authors:** Siyang Liu, Qian Chen, Jiayang Li, Yimeng Zhu, Xiaorong Guo, Xin Zhao

**Affiliations:** ^1^ Key Laboratory of Adolescent Cyberpsychology and Behavior (CCNU) Ministry of Education Wuhan China; ^2^ Key Laboratory of Human Development and Mental Health of Hubei Province School of Psychology Central China Normal University Wuhan China; ^3^ Manchester Institute of Education The University of Manchester Manchester UK

**Keywords:** adolescent smartphone use, item response theory, network analysis, problematic smartphone use, psychometric

## Abstract

**Objectives:**

This study assessed the psychometric properties of the Smartphone Application‐Based Addiction Scale (SABAS) among Chinese primary school students, focusing on validity, reliability, and factor structure using Item Response Theory (IRT) and Network Analysis (NA).

**Methods:**

Data were collected from 1108 primary school students in China (52.98% female; ages 7–14 years; *M* = 10.58, SD = 0.99). SABAS was assessed using Item Response Theory (IRT) for factor structure, item parameters, cut‐off scores, and reliability, while Differential Item Functioning (DIF) detected gender biases. Network Analysis (NA) examined the interrelationships among SABAS items.

**Results:**

Confirmatory factor analysis supported SABAS's unidimensional structure (RMSEA = 0.055, CFI = 0.984, TLI = 0.973, SRMR = 0.025). IRT indicated high item discrimination (*α* = 1.47–2.47) and identified a cut‐off score of 27, classifying 1.7% of students as high‐risk for problematic smartphone use. Gender DIF was noted in item 6, with boys showing higher relapse tendencies (*p* < 0.05). NA highlighted the centrality of tolerance and withdrawal items.

**Conclusions:**

SABAS is a reliable tool for assessing problematic smartphone use in Chinese primary school students, particularly those at moderate to high risk.

## 1 Introduction

Smartphone use has become widespread among children and adolescents, who depend heavily on various applications for daily needs (Council on Communications and Media 2016). However, this reliance can lead to excessive and compulsive use, negatively impacting mental health.

In China, PSU is a significant behavioral concern affecting a substantial number of adolescents (Chang and Ko 2023). A review estimated that the prevalence of PSU among adolescents ranges from 14.0% to 31.2% (Sohn et al. 2019). Factors such as age and gender may influence the prevalence of PSU, although findings remain inconsistent. For instance, some studies indicate that female adolescents are more prone to PSU than their male counterparts (Emirtekin et al. 2019; C. Lee and Lee 2017), while others report the opposite (Kwak et al. 2018; Soni et al. 2017). Similarly, while some research suggests that older adolescents exhibit higher rates of PSU (Fırat et al. 2018; E. J. Lee and Ogbolu 2018), while others found no significant age difference or a reverse trend (Ayar et al. 2017; Lopez‐Fernandez et al. 2014).

To evaluate the prevalence and severity of PSU, researchers have developed various psychometric tools across cultural contexts (Harris et al. 2020). Among these, the Smartphone Application‐Based Addiction Scale (SABAS) stands out for its brevity and widespread use. Originally developed by Csibi et al. (2018) based on Griffiths' addiction components model (Griffiths 2005), the SABAS includes six items that assess key aspects of behavioral addiction: salience, mood modification, tolerance, withdrawal, conflict, and relapse. This scale employs ordered polytomous items to capture variations in PSU effectively. The SABAS has shown good reliability and validity across diverse populations, including English (Mason et al. 2022), Persian (Lin et al. 2019), Italian (Soraci et al. 2020), Serbian (Vujić et al. 2023), Indonesian (Nurmala et al. 2022), and Hong Kongese samples (Yam et al. 2018). Consequently, this study utilizes the SABAS to assess PSU among Chinese primary school students.

Currently, research on the psychometric properties of the SABAS in this population is limited. Chinese primary school students are particularly susceptible to PSU due to their early and frequent exposure to smartphones (Fischer‐Grote et al. 2019). Additionally, many existing studies have relied on classical test theory (CTT) to evaluate the SABAS (Mason et al. 2022; Soraci et al. 2020; Vujić et al. 2023; Yam et al. 2018). However, CTT has inherent limitations, including sample dependence, score dependence, and a lack of precision at varying levels of the latent trait (Liu et al. 2020).

In contrast, Item Response Theory (IRT) offers a more nuanced approach to psychometric evaluation. IRT models the relationship between an individual's latent traits (i.e., the level PSU) and their item responses, allowing for a detailed analysis of item characteristics and person parameters (Embretson and Reise 2013). This theory provides advantages such as invariant item parameters across different populations and the ability to assess item performance at varying levels of ability (Embretson and Reise 2013), making it particularly suitable for evaluating scales like the SABAS.

Furthermore, Network Analysis presents a contemporary framework for understanding the relationships between variables in complex systems (Epskamp et al. 2018). In the context of psychological constructs, it allows researchers to visualize and assess the interconnections among symptoms and factors related to PSU (Poetar et al. 2024). By representing these relationships as networks, researchers can identify key drivers of PSU and understand how different elements influence each other (Huang et al. 2021; Poetar et al. 2024). This method enhances our understanding of the multifaceted nature of behavioral addiction, offering insights into potential intervention points.

Therefore, this study aims to address these gaps by employing innovative approaches such as item response theory (IRT) and network analysis to investigate the SABAS in a large sample of Chinese primary school students.

## 2 Methods

### 2.1 Participants and Procedure

The initial sample consisted of 1150 students in grades four to six from three primary schools in Sichuan province of China. After removing incomplete or inconsistent responses, valid data were obtained from 1108 participants, resulting in a response rate of 96.35%. Among the respondents, 52.98% were female, and their ages ranged from 7 to 14 years (*M* = 10.58, SD = 0.99). Informed consent was secured from both the students and their parents. Students completed survey scales along with a background questionnaire that included gender, age, and grade level. Ethics approval was granted by the lead author's institution, and participants were assured of their anonymity and their right to withdraw from the study at any time.

### 2.2 Measures

#### 2.2.1 Smartphone Application‐Based Addiction Scale (SABAS, Csibi et al. 2018)

The SABAS has been translated into Chinese (Chen et al. 2020) and underwent several translation protocols, including back‐translation, modification of word meaning, and cultural sensitivity checks to ensure readability. The SABAS is a six‐item scale designed to assess the risk of PSU in participants, based on the addiction components model (i.e., salience, mood modification, tolerance, withdrawal, conflict, and relapse) (Griffiths 2005). Participants indicate their agreement with each item (e.g., “My phone is the most important thing in my life”) on a five‐point Likert scale ranging from 1 (*strongly disagree*) to 5 (*strongly agree*). Total scores range from 6 to 30, with higher scores indicating a greater degree of PSU risk. The scale demonstrated good internal reliability in the present study (Cronbach's *α* = 0.81, McDonald's *ω* = 0.83).

#### 2.2.2 Nine‐Item Internet Gaming Disorder Scale‐Short Form (IGDS9‐SF) (Pontes and Griffiths 2015)

The IGDS9‐SF is a scale based on the nine criteria for internet gaming disorder (IGD) in the Diagnostic and Statistical Manual of Mental Disorders Fifth Edition (DSM‐V). It measures the risk of developing internet gaming disorder using a 5‐point Likert scale from 1 (never) to 5 (very often) for each item. A higher score indicates a higher risk of developing IGD. The scale has a unidimensional structure and a high internal consistency (Cronbach's *α* = 0.90).

#### 2.2.3 Bergen Social Media Addiction Scale (BSMAS) (Andreassen et al. 2016)

The BAMAS is a scale that uses the six components of the addiction model (i.e., salience, mood, modification, tolerance, withdrawal, conflict, and relapse) (Griffiths 2005) to assess the risk of social media addiction. It consists of six items on the use of social media in the past year. Each item is rated on a 5‐point Likert scale from 1 (very rarely) to 5 (very often). A higher BSMAS score suggests a higher risk of social media addiction. The BSMAS has a unidimensional structure and good psychometric properties, such as internal consistency (Cronbach's *α* = 0.82).

### 2.3 Statistical Analyses

Firstly, exploratory factor analysis (EFA) and confirmatory factor analysis (CFA) were performed to examine the factor structure of the SABAS. EFA was conducted using the R package *psych* (Revelle 2017), with the Reckase criterion used to determine the proportion of variance explained by the first factor, ensuring that it exceeded 20% and that the ratio of variance between the first and second factors was greater than 4 (Liu et al. 2020, 2023). CFA was conducted using the R package lavaan (Rosseel 2012), with fit indices indicating a close fit if the root mean square error of approximation (RMSEA) < 0.08, standardized root mean square residual (SRMR) < 0.08, the comparative fit index (CFI) > 0.90, and Tucker‐Lewis Index (TLI) > 0.90 (Browne and Cudeck 1993). Moreover, the concurrent validity was tested using Pearson correlation between the SABAS, BSMAS, and IGDS9‐SF.

Subsequently, IRT analyses were then applied to assess the psychometric properties of the SABAS. At the item level, IRT was used to estimate item parameters, including discrimination (*α*) and difficulty (*β*), as well as the item information function (IIF), which reflects measurement precision at different levels of the latent trait (Liu et al. 2023). At the test level, the test characteristic curve (TCC) and test information function (TIF) were used to evaluate the overall reliability of the scale in measuring PSU (Samajima 1994). Moreover, SABAS raw scores were converted into PSU levels (*θ*) based on the summed scale expected a posteriori scores (SSEAP [*θ*|*x*]), and participants scoring above +2SD were classified as high‐risk (Thissen et al. 1995; Zarate et al. 2022). Age and gender differences in PSU were assessed using Welch's *t*‐test and *χ* ^2^ tests.

Differential Item Functioning (DIF) analysis was conducted to assess whether SABAS items functioned equivalently across genders. A logistic regression (LR) method was used to detect DIF items, with the Benjamini‐Hochberg procedure controlling for Type I errors in multiple comparisons (Swaminathan and Rogers 1990). The LR analysis was performed using *R* package *mirt* (Chalmers 2012).

Finally, Network Analysis examined relationships between variables using a regularized partial correlation network based on the Gaussian graphical model (Costantini et al. 2015). The network was estimated with the EBICglasso method for ordered categorical variables (Epskamp et al. 2018). The EBICglasso method was chosen due to its ability to penalize weaker associations, ensuring a parsimonious model that avoids overfitting, particularly when analyzing high‐dimensional psychometric data. Edges close to zero were excluded via the least absolute shrinkage and selection operator (LASSO; Tibshirani 1996). The analysis utilized the R package *bootnet* (Epskamp and Fried 2015) and was visualized with *qgraph* (Epskamp et al. 2012).

Centrality indices were used to assess the importance of nodes in the network, with Expected Influence (EI) being preferred over Strength due to its capability to consider negative edges (Robinaugh et al. 2016). Other centrality indices, such as Closeness and Betweenness, were not used due to criticisms regarding their reliability (Bringmann et al. 2019). EI was plotted using qgraph, while the normalized proportion of correct classification (nCC) for categorical variables was derived from mgm (Haslbeck and Waldorp 2020) and displayed as pie charts on node borders.

Edge weight accuracy was evaluated using 95% bootstrap confidence intervals (Epskamp et al. 2018) with wider intervals indicating lower accuracy. The stability of edges and EI values was assessed via the correlation stability coefficient (CS‐coefficient), with values greater than 0.50 indicating acceptable stability. These analyses were conducted with *bootnet* (Epskamp and Fried 2015).

## 3 Results

### 3.1 Factor Structure and Concurrent Validity

To confirm the unidimensional structure of SABAS and to prepare for the IRT analysis, EFA and CFA were performed. In SABAS, the first essential eigenvalue was 3.12, which was substantially higher than the second eigenvalue of 0.74, with the ratio of the first to the second eigenvalue exceeding 4. The scree plot (see Supporting Information S1: Figure S1) clearly showed that the curve flattens out after the first component. Moreover, the first factor accounted for over 50% of the variance, surpassing the 20%. In addition, the goodness‐of‐fit indices from the single‐factor CFA model (RMSEA = 0.055; SRMR = 0.025; CFI = 0.984; TLI = 0.973) suggested a good fit. All item factor loadings were positive, ranging from 0.55 to 0.72 (see Supporting Information S1: Table S1). Furthermore, the concurrent validity of the SABAS was supported by significant correlations with the BSMAS total score (*r* = 0.554; *p* < 0.001) and the IGDS9‐SF total score (*r* = 0.694; *p* < 0.001) (see Supporting Information S1: Table S2).

### 3.2 IRT Parameter Estimation

**FIGURE 1:**
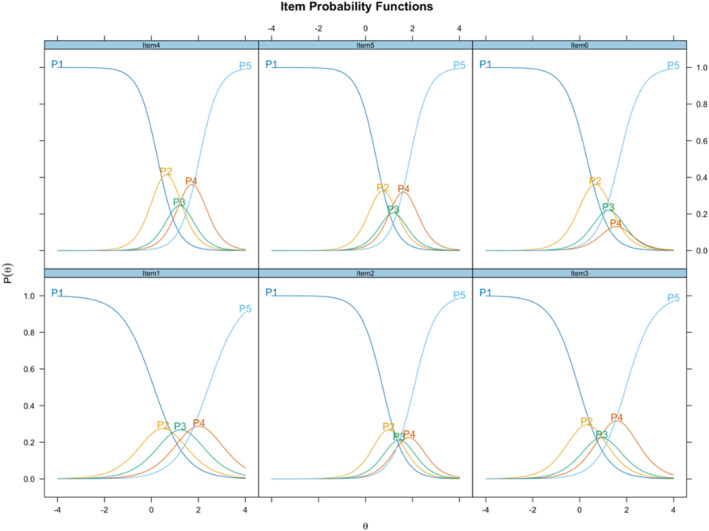
SABAS item characteristic curves (ICCs). Here, theta (*θ*) represents latent trait levels, and probability indicates the likelihood of endorsing an item based on difficulty (*β*) and latent trait level.

As shown in Table 1, all items demonstrated high discrimination (*α*) capacity (0.65–1.34 = moderate; 1.35–1.69 = high; greater than 1.70 = very high) (Barak 2011). The items in descending order of *α* were 1, 3, 6, 2, 4, and 5. Considering difficulty (*β*) thresholds, there were fluctuations between the different thresholds (see Figure 1). For example, while the ascending sequence of *β* for the first threshold (*β*_1_‐*strongly disagree*) is Item 3 (Mood modification), Item 4 (Tolerance), Item 6 (Relapse), Item 5 (Withdrawal), Item 2 (Conflicts), and Item 1 (Salience), the ascending sequence of *β* for the last threshold (*β*_5_‐*strongly agree*) was Items 6, 5, 3, 4, 2, and 1.

**TABLE 1:** Item discrimination and difficulty parameters of the SABAS.

Item	Addiction component	*α*	*β*_1_	*β*_2_	*β*_3_	*β*_4_
1	Salience	1.47	0.10	0.87	1.62	2.42
2	Conflicts	2.16	0.73	1.24	1.64	2.06
3	Mood modification	1.71	−0.04	0.67	1.20	1.97
4	Tolerance	2.42	0.27	1.00	1.41	2.03
5	Withdrawal	2.47	0.46	1.01	1.35	1.88
6	Relapse	2.14	0.31	1.02	1.44	1.69

*Note: α* (discrimination) = the capacity of an item to discriminate between varying levels of the behavior intensity (*θ*). *β* (difficulty thresholds) = the level of behavior intensity, where subsequent response rates are more probable than their previous rate.

**FIGURE 2:**
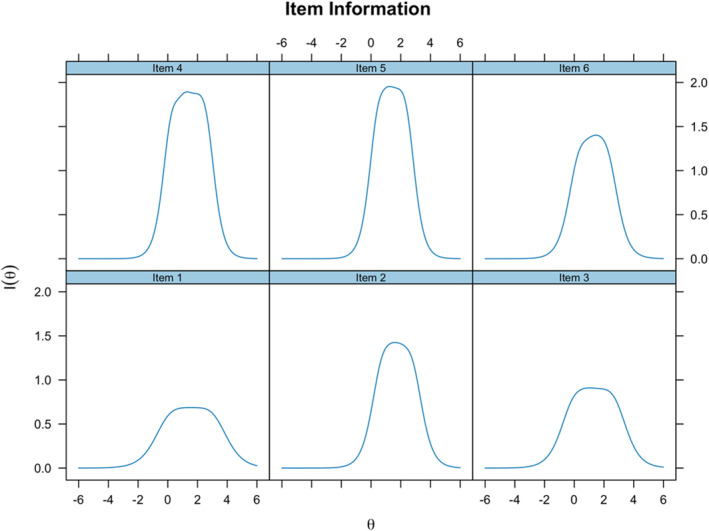
SABAS item information function (IIF). These figures demonstrate how reliability indices vary at different *θ* levels. These indices are conditional upon stander errors, with increased errors representing lower reliability.

Meaningful differences were observed in item information, which is analogous to measurement reliability, across different levels of the latent trait *θ* (see Figure 2). Item 5 provided the highest level of information between +0.2 SD and +2.1 SD, followed closely by Item 4, which provided a high level of information between 0 and +2.5 SD. Items 2 and 6 were similar, providing good levels of information between 0 and +3 SD. Items 1 and 3 showed acceptable reliability above −1 SD, but very limited reliability below this threshold. More specifically, Item 3 was most reliable between −1 SD and +3 SD, while Item 1 was most reliable above +3 SD.

### 3.3 IRT Properties at Scale Level and Prevalence

**FIGURE 3:**
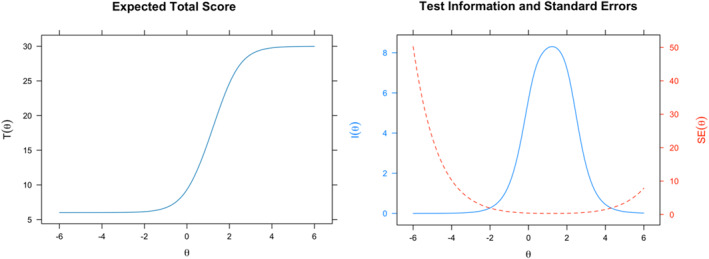
SABAS test characteristic curve (TCC; left panel) and test information curve (TIC; right panel). The TCC illustrates the good overall performance of the BSMAS as a scale, with PSU increasing as SABAS score increase. The TIC illustrates the conditional effect of standard measurement error (SEM; dotted line) on reliability indices, with increased reliability for reduced SEM.

Considering the SABAS scale as a whole, the test characteristic curve (TCC) and test information function (TIF) collectively demonstrate the scale's performance and reliability across all six items. The TCC shows a steep increase in SABAS scores as the total reported PSU score rises, with the most significant increase occurring between scores of 8 and 28. The TIF indicates sufficient reliability and information between −2 SD and +4 SD, peaking between +0.5 SD and +2.2 SD. Using IRT‐based conversion (SSEAP [*θ*|*x*]), raw SABAS scores of 14, 18, and 27 correspond to +0.5, +1, and +2 standard deviations (SD) on the scale, respectively (see Supporting Information S1: Table S3). Therefore, a score of 27 or higher may serve as a provisional diagnostic cut‐off for PSU risk, with 1.7% of participants (*n* = 19) meeting this criterion (Figure 3).

### 3.4 The PSU and Gender/Age

A *χ* ^2^ test of independence was used to investigate the relationship between binary genders (521 males, 587 females) and PSU (19 high‐risk, 1089 non‐high‐risk). No significant differences in high‐risk for PSU were observed between males and females (*χ* ^2^ = 0.244, *p* = 0.621). More specifically, 0.81% of females (*n* = 9) had PSU scores in the range of the conditional cut‐off score (≥ 27), compared to 0.90% of male (*n* = 10). However, a Welch's independent sample *t*‐test detected significant differences in SABAS scores across gender groups (*t* = 4.908, *p* < 0.001, [95% CI = 0.948, 2.210], Cohen's *d* = 0.30) with males scoring higher (*M* = 11.94) than females (*M* = 10.36).

In addition, a Welch's independent sample *t*‐test was used to compare age differences and PSU (high‐risk vs. non‐high‐risk). Results indicate that the high‐risk PSU group (*M*_age_ = 10.84) was not significantly younger than the non‐high‐risk group (*M*_age_ = 10.58) with a small size effect (*t* = 1.270, *p* = 0.220, [95% CI = −0.172, 0.701], Cohen's *d* = 0.27).

### 3.5 Differential Item Functioning Analysis

Using the iterative purification procedure, the SABAS items were used for testing DIF. Any SABAS item with *χ* ^2^ associated probability less than the B‐H adjusted overall alpha level of 0.05 was flagged as DIF.

In this study, gender DIF was identified on the item discrimination parameters (*α*) on item 6 (*p* < 0.05) “If I try to cut the time I use my smartphone, I manage to do so for a while, but then I end up using it as much or more than before” in favor of males. In other words, the item exhibited higher discrimination power for boys than girls (see Supporting Information S1: Figure S2). Among all studied items, no significant differences in the item severity parameters (*β*) across genders were detected, suggesting that the SABAS items function equally for boys and girls in terms of item severity.

### 3.6 Network Analysis

**FIGURE 4:**
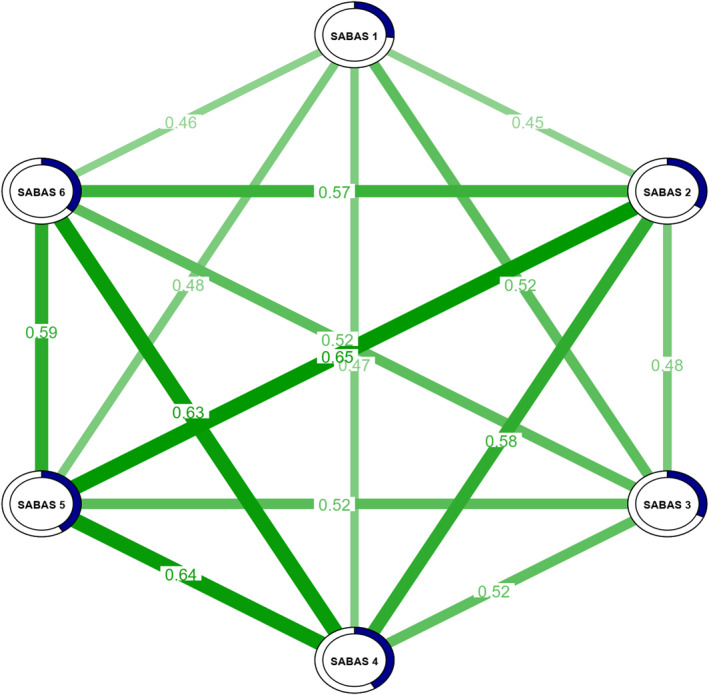
Network model estimated at the level of SABAS items. Green edges stand for a positive connection. The thickness of the lines is proportional to edge weight strength. The values on the lines represent partial correlation coefficients. The pie charts bordering the nodes represent nCC values.

The network estimated at the level of SABAS items is presented in Figure 4. The stability of edge weights and centrality indices was excellent, with a CS coefficient of 0.60 for both edge weights and Expected Influence (EI) indices. However, edge weight accuracy was relatively low, as indicated by the large 95% bootstrap confidence intervals (seen in Supporting Information S1: Figure S3). The stability of the EI index is further supported by the high average correlation with the original sample across different sampled cases, demonstrating reliable stability even as the sample size decreased to 30% of the original cases (seen in Supporting Information S1: Figure S4).

**FIGURE 5:**
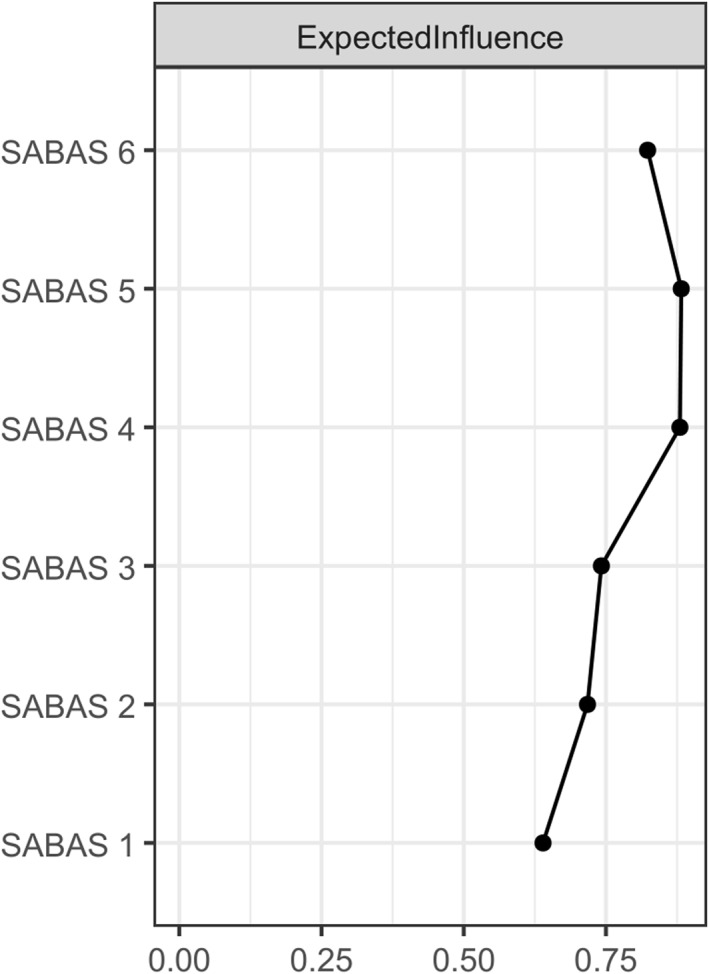
Expected influence (EI) values for individual SABAS items. The plot shows the expected influence for each item in the SABAS network, indicating the relative impact of each item within the network structure.

As shown in Figure 5, the symptoms with the highest EI were item 5 (Withdrawal), which was stronger than all other nodes, and item 4 (Tolerance), which was stronger than 67% of the nodes. The strongest edge was between item 2 (Conflicts) and item 5 (Withdrawal), surpassing all other edges in the model. Additionally, the edge linking item 4 (Tolerance) and item 5 (Withdrawal) was significantly stronger than 74% of the other edges. The nCC ranged from 0.263 for item 1 to 0.410 for item 5 and 0.414 for item 4, indicating that 41.0% of the variance in Withdrawal and 41.4% in Tolerance could be explained by the nodes they were connected to.

## 4 Discussion

The present study contributes significantly to the literature on problematic smartphone use (PSU) among primary school students by employing a combination of Item Response Theory (IRT) and Network Analysis (NA) to evaluate the psychometric properties of the SABAS. Our findings demonstrate that the SABAS is a valid and reliable tool for assessing PSU in this population, particularly in identifying those at moderate to high risk. These results align with recent research emphasizing the need for reliable psychometric tools for assessing PSU across various cultural contexts (Lu et al. 2024; Poetar et al. 2024).

### 4.1 Insights From IRT Analysis

Using IRT, we provided detailed information on item and test characteristics, including discrimination, difficulty, information, reliability, and cut‐off scores. The summed score expected a posteriori (SSEAP [*θ*|*x*]) method was employed to estimate prevalence and identify an optimal threshold for high‐risk PSU based on IRT results. Notably, only 1.7% of participants were classified as high‐risk for PSU. This low prevalence may be attributed to the socio‐cultural and regulatory environment in China, where there is a strong emphasis on academic achievement, and parents and schools enforce strict technology use regulations, particularly for younger children. Measures such as restricted usage times, content monitoring, and bans during school hours help to limit exposure and promote disciplined technology use. These controls likely mitigate the risk of PSU in this population. The low prevalence also underscores the importance of early intervention and preventive measures tailored to the specific needs of primary school students. It suggests that appropriate guidance and regulation can effectively manage the risk of PSU, even among groups considered vulnerable due to early and high exposure to smartphones.

We also examined the relationship between PSU and gender and age, which are important factors that may influence smartphone use patterns and outcomes. Contrary to some previous studies that reported significant associations between female gender and problematic usage (Emirtekin et al. 2019; C. Lee and Lee 2017), or higher scores in boys compared to girls (Kwak et al. 2018; Soni et al. 2017) we found no significant gender differences in PSU prevalence or severity in our sample. This suggests that gender differences in PSU may not be universal or consistent across different age groups or cultural contexts. However, we did find gender DIF on item 6, which measures relapse tendency, indicating that boys were more likely than girls to endorse this item at the same latent trait level. This suggests that boys may experience greater difficulty in controlling smartphone use once they attempt to reduce it, consistent with previous research showing higher impulsivity and lower self‐regulation in boys (Choi et al. 2015).

### 4.2 Insights From Network Analysis

Network Analysis offered additional insights into the internal structure of the SABAS and the relationships among its items. The analysis revealed that all items were positively correlated, supporting the conceptualization of PSU as a coherent syndrome (Busch and McCarthy 2020; Wacks and Weinstein 2021). By quantifying the centrality of symptoms, we gained a more nuanced understanding of their contributions to PSU.

Withdrawal (item 5) and Tolerance (item 4) were identified as the most central nodes, indicating their key role in maintaining the PSU network. This aligns with Griffiths' (2005) components model of addiction, which emphasizes tolerance and withdrawal as core features of behavioral addiction. Our findings validate the significance of these components within PSU, consistent with studies on smartphone‐related behavioral addictions (Huang et al. 2021). These results suggest that targeting Withdrawal and Tolerance through interventions could have a cascading effect in reducing other symptoms, as interventions focusing on central symptoms tend to disrupt the overall network structure (Borsboom and Cramer 2013).

The strong link between Conflicts (item 2) and Withdrawal (item 5) highlights that interpersonal conflicts are closely tied to withdrawal symptoms, aligning with prior research on social difficulties and PSU (Bian and Leung 2015). Studies further indicate that social isolation amplifies the emotional impact of smartphone withdrawal, increasing stress and anxiety (Elhai et al. 2020). This underscores the importance of addressing both individual behaviors and social interactions in interventions, such as incorporating social skills training and conflict resolution to reduce dependence on smartphones.

### 4.3 Limitations and Future Directions

Despite the valuable insights provided, this study has several limitations. First, its cross‐sectional design precludes causal inferences. Longitudinal studies are needed to better understand the developmental trajectory of PSU and identify causal pathways. Second, our reliance on self‐report measures may introduce bias, including social desirability and recall errors. Future research should incorporate objective measures, such as smartphone usage logs, to validate the findings. Third, the study focused exclusively on primary school students in China, which may limit the generalizability of the results. Further studies should include more diverse populations to determine the broader applicability of the SABAS and its psychometric properties.

Lastly, while our findings highlight the potential impact of parental and school regulations on reducing PSU, further empirical validation of these interventions is required. Future research should explore the efficacy of different types of parental controls and educational policies in mitigating PSU among children and adolescents.

## Author Contributions

**Siyang Liu:** data curation, formal analysis, investigation, methodology, validation, writing – original draft. **Qian Chen:** data curation, investigation, methodology, writing – review and editing. **Jiayang Li:** data curation, formal analysis, investigation. **Yimeng Zhu:** Data curation, formal analysis. **Xiaorong Guo:** conceptualization, methodology, project administration, writing – review and editing. **Xin Zhao:** conceptualization, methodology, project administration, writing – review and editing.

## Ethics Statement

The research protocol underwent a thorough evaluation and received approval from the Research Ethics Committee of Central China Normal University (Ethics approval number: CCNU‐IRB‐202201020). The study was conducted in accordance with the Declaration of Helsinki and other relevant ethical guidelines, ensuring respect for participants' rights and privacy.

## Consent

Written parental consent was obtained from the parents or legal guardians of the participants before the study commenced.

## Conflicts of Interest

The authors declare no conflicts of interest.

## Supporting information

Supporting Information S1

## Data Availability

The data that support the findings of this study are available in the Figshare repository (https://figshare.com/s/0728022a08b5d5269863).
