# Supplementary material for: Evaluating Problematic Smartphone Use Among Chinese Primary School Students Using SABAS: An IRT and Network Analysis
Source: Int J Methods Psychiatr Res. 2025 Apr 1;34(2):e70016. doi: 10.1002/mpr.70016 (PMC11959412; doi:10.1002/mpr.70016)

**APPENDIX:**

*Evaluating Problematic Smartphone Use Among Chinese Primary School Students Using SABAS: An IRT and Network Analysis*

**Table S1.** The CFA item loadings on the SABAS.

| Item | Label | λ | *SE* | *Z* | Stand. λ |
| --- | --- | --- | --- | --- | --- |
| 1 | Salience | 1 |  |  | 0.551 |
| ­­2 | Tolerance | 1.078 | 0.07 | 15.301 | 0.645 |
| 3 | Mood modification | 1.196 | 0.081 | 14.819 | 0.612 |
| 4 | Relapse | 1.244 | 0.076 | 16.303 | 0.723 |
| 5 | Withdrawal | 1.284 | 0.079 | 16.246 | 0.718 |
| 6 | Conflict | 1.258 | 0.081 | 15.599 | 0.666 |

SABAS= Smartphone Application-Based Addiction Scale. *SE* = Standard Error. λ = factor loading estimates and standardized estimates. All item loadings on the single latent factor (social media addiction) were significant (*p* < .001). *Z* = item standard score.

**Table S2.** Pearson Correlation Among SABAS, BSMAS, and IGDS-SF9 Scores.

|  | SABAS | BSMAS | IGDS-SF9 |
| --- | --- | --- | --- |
| SABAS | 1 |  |  |
| BSMAS | 0.554^**^ | 1 |  |
| IGDS-SF9 | 0.694^**^ | 0.549^**^ | 1 |

SABAS= Smartphone Application-Based Addiction Scale. BSMAS = Bergen Social Media Addiction Scale. IGDS9-SF = Internet Gaming Disorder Scale-Short Form.

^**^ Statistically significant at *p* < .001

| Table S3. Summed SABAS score to scale score conversion based on expected a posteriori distribution | | | |
| --- | --- | --- | --- |
| Summed score | EAP[θ\|x] | SD[θ\|x] | Modelled proportion |
| 6 | -1.117 | 0.644 | 0.257 |
| 7 | -0.539 | 0.495 | 0.079 |
| 8 | -0.294 | 0.447 | 0.073 |
| 9 | -0.051 | 0.405 | 0.088 |
| 10 | 0.057 | 0.395 | 0.078 |
| 11 | 0.248 | 0.370 | 0.054 |
| 12 | 0.356 | 0.363 | 0.048 |
| 13 | 0.457 | 0.363 | 0.051 |
| 14 | 0.578 | 0.362 | 0.041 |
| 15 | 0.730 | 0.347 | 0.042 |
| 16 | 0.814 | 0.346 | 0.029 |
| 17 | 0.940 | 0.357 | 0.023 |
| 18 | 1.064 | 0.335 | 0.021 |
| 19 | 1.168 | 0.338 | 0.019 |
| 20 | 1.248 | 0.350 | 0.026 |
| 21 | 1.357 | 0.347 | 0.013 |
| 22 | 1.480 | 0.336 | 0.017 |
| 23 | 1.559 | 0.334 | 0.007 |
| 24 | 1.674 | 0.346 | 0.006 |
| 25 | 1.811 | 0.363 | 0.007 |
| 26 | 1.852 | 0.329 | 0.004 |
| 27 | 2.106 | 0.383 | 0.007 |
| 28 | 2.241 | 0.383 | 0.005 |
| 29 | 2.361 | 0.398 | 0.002 |
| 30 | 2.717 | 0.479 | 0.003 |
| *Note.* SABAS= Smartphone Application-Based Addiction Scale. While ‘summed scores’ represent raw SABAS scores, EAP (expected a posteriori; EAP[θ\|x]) scores estimate the mean of the posterior distribution of θ, given a patterned response to x. A measure of the EAP precision can be observed by the posterior standard deviation (SD[θ\|x]). The modelled proportion represent the population distribution likely to obtain a determined raw score based on EAP[θ\|x]. | | | |

**Figure S1** The scree plot of the extracted factors from the real response data set


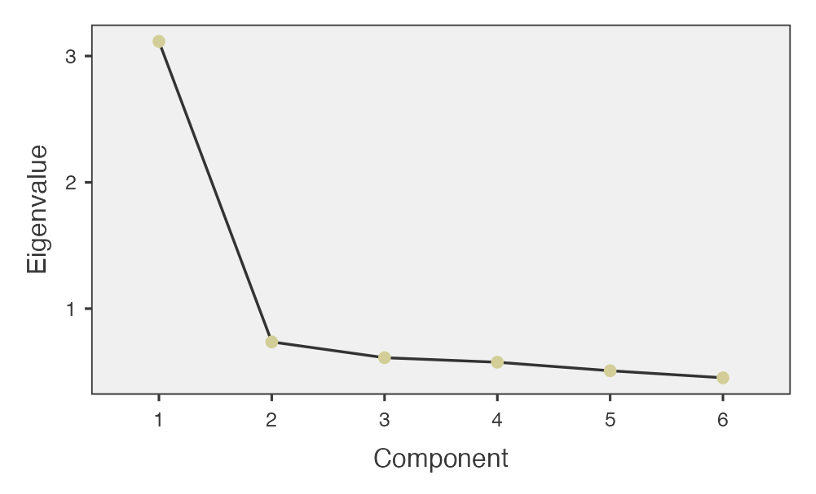


**Figure S2** The item characteristic curves on 5 response categories of Item 6.


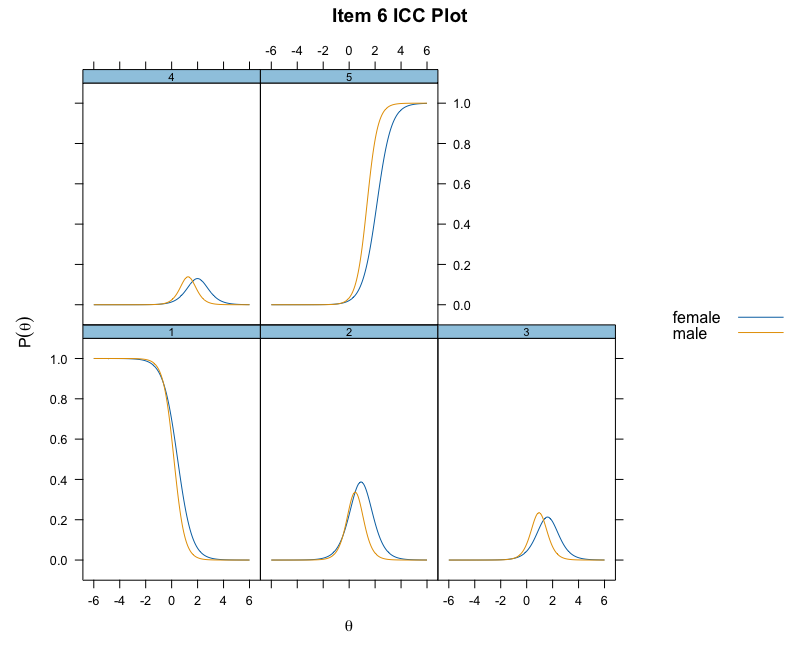


**Figure S3** Bootstrapped confidence intervals of estimated edge-weights for the estimated network of SABAS items. The *red line* indicates the sample values and the *gray area* the bootstrapped CIs. Each horizontal line represents one edge of the network, ordered from the edge with the highest edge-weight to the edge with the lowest edge-weight.


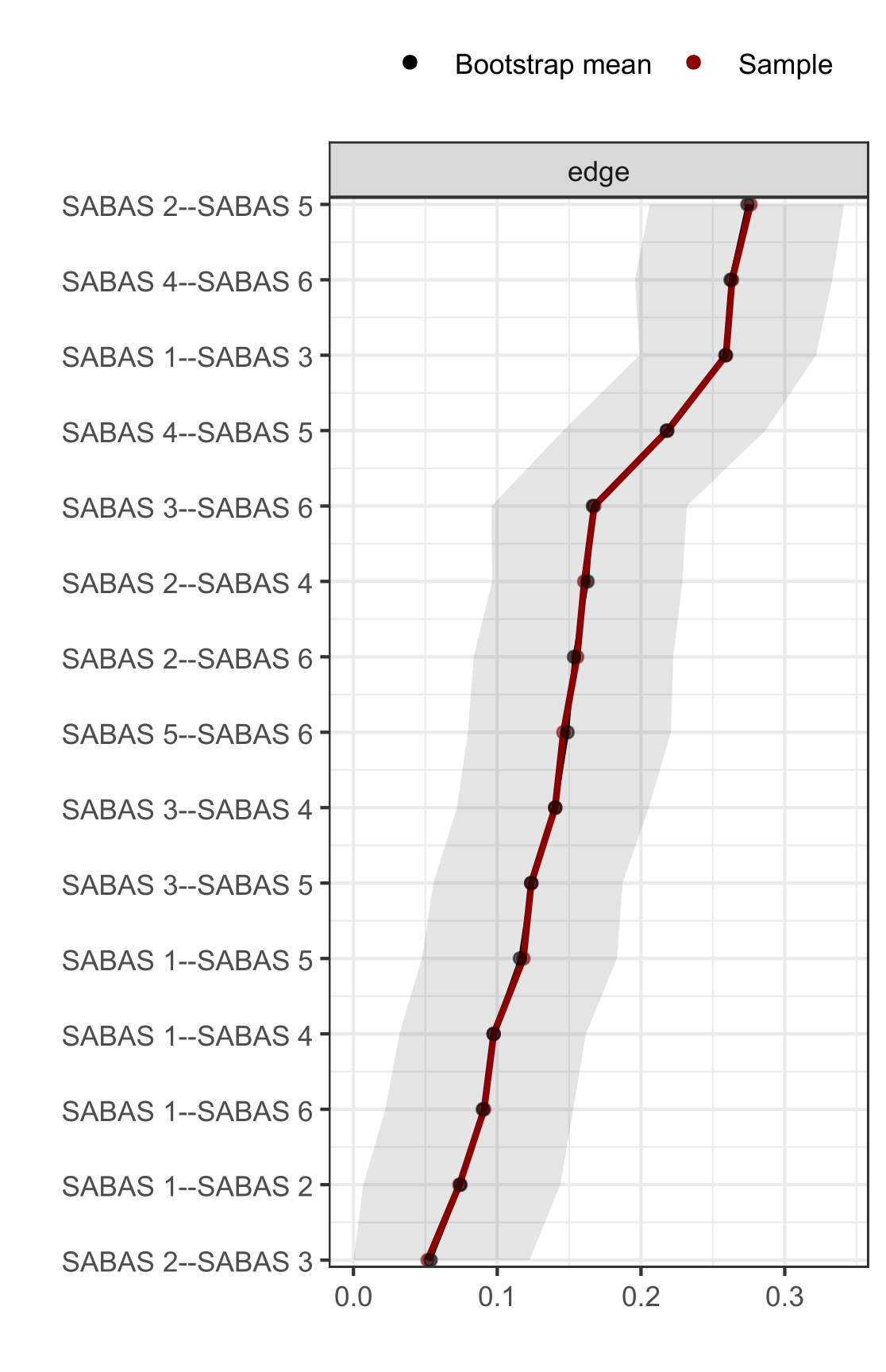


**Figure S4** Correlation stability plot of Expected Influence for the estimated network of SABAS items. The red line indicates the average correlation with the original sample as different percentages of cases are sampled, and the shaded area represents the 95% confidence interval.


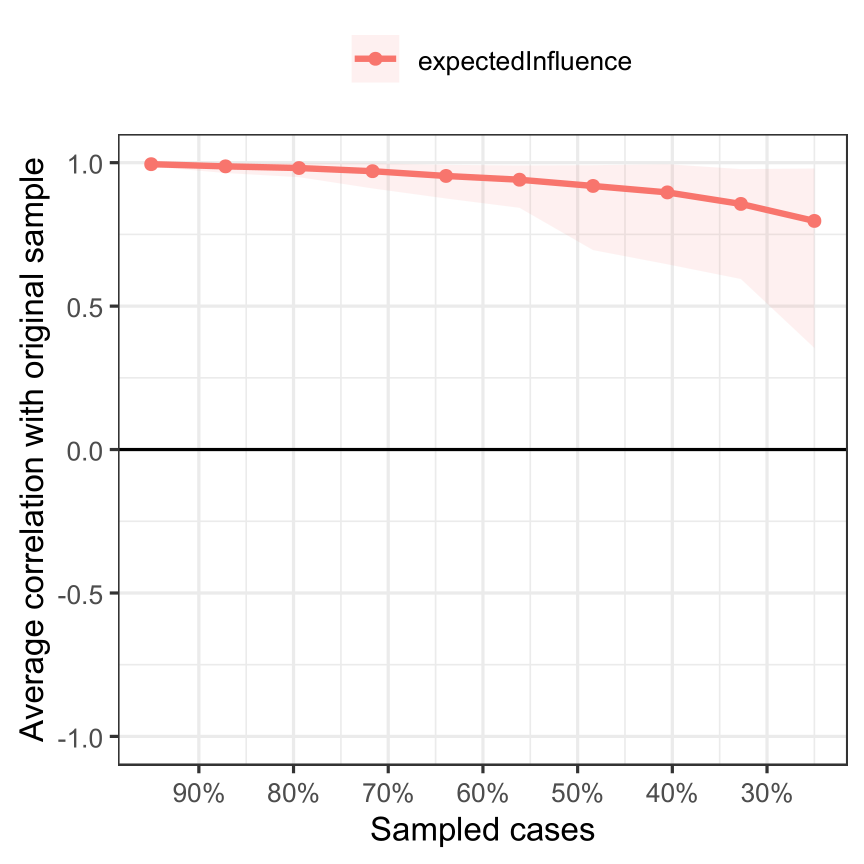

Supplement: Supplementary file 1 — Supporting Information S1 [file MPR-34-e70016-s001.docx]
